# Gas-Phase Fabrication and Photocatalytic Activity of TiO_2_ and TiO_2_–CuO Nanoparticulate Thin Films

**DOI:** 10.3390/ma17051149

**Published:** 2024-03-01

**Authors:** Meditha Hudandini, Kusdianto Kusdianto, Masaru Kubo, Manabu Shimada

**Affiliations:** 1 Chemical Engineering Program, Graduate School of Advanced Science and Engineering, Hiroshima University, 1-4-1, Kagamiyama, Higashi-Hiroshima, Hiroshima 739-8527, Japan; d213956@hiroshima-u.ac.jp (M.H.); mkubo@hiroshima-u.ac.jp (M.K.); 2 Department of Chemical Engineering, Institut Teknologi Sepuluh Nopember (ITS), Kampus ITS Sukolilo, Surabaya 60111, Indonesia; kusdianto@chem-eng.its.ac.id

**Keywords:** plasma-enhanced chemical vapor deposition, physical vapor deposition, photodecomposition, H_2_O_2_, rhodamine 6G

## Abstract

CuO-loaded TiO_2_ nanomaterials have applications in pollutant degradation via photocatalysis. However, the existing methods of fabricating these nanomaterials involve liquid-phase processes, which require several steps and typically generate liquid waste. In this study, TiO_2_ and TiO_2_–CuO nanoparticulate thin films were successfully fabricated through a one-step gas-phase approach involving a combination of plasma-enhanced chemical vapor deposition and physical vapor deposition. The resulting films consisted of small, spherical TiO_2_ nanoparticles with observable CuO on the TiO_2_ surface. Upon annealing in air, the TiO_2_ nanoparticles were crystallized, and CuO was completely oxidized. The photocatalytic activity of TiO_2_–CuO/H_2_O_2_, when introduced into the rhodamine 6G degradation system, was substantially enhanced under both ultraviolet and visible light irradiation. Moreover, this study highlights the influence of pH on the photocatalytic activity; TiO_2_–CuO/H_2_O_2_ exhibited the highest photocatalytic activity at pH 13, with a reaction rate constant of 0.99 h^−1^ cm^−2^ after 180 min of visible light irradiation. These findings could facilitate the development of nanoparticulate thin films for enhanced pollutant degradation in wastewater treatment.

## 1. Introduction

Human activities have intensified the environmental challenges associated with pollution and the availability of clean water. To address water pollution, advanced oxidation processes (AOPs), such as photocatalysis, have been reported to be more effective than conventional wastewater treatment methods, such as adsorption, flocculation, and coagulation, which have higher operating costs [1,2,3]. Photocatalysis involves the creation of highly reactive hydroxyl radicals that are pivotal to the partial or complete mineralization of organic pollutants [3,4]. Extensive research has been conducted to investigate functional semiconductor nanomaterials that can oxidize these pollutants through photocatalysis.

TiO_2_ is a key material in photocatalytic applications owing to its thermal and chemical stability, nontoxicity, cost-effectiveness, and availability [5,6,7]. The anatase phase of TiO_2_ exhibits higher photocatalytic activity than the rutile and brookite phases. However, it still has some disadvantages because of its wide bandgap (3.2 eV), which suppresses the photoactivity owing to rapid electron–hole recombination [3,5,8] and limits light absorption primarily to the ultraviolet (UV) region [8], which constitutes only 5% of the solar spectrum.

Various transition metal oxide semiconductors such as WO_3_, ZnO, SiO_2_, Fe_2_O_3_, and CuO [2,9] have been incorporated into TiO_2_ to enhance its activity. In this regard, economical, environmentally safe, abundant, and highly active copper oxides (Cu^2+^ and Cu^1+^) are suitable for synergistic integration with TiO_2_ [5,7,10]. TiO_2_ (an n-type semiconductor) and CuO (a p-type semiconductor) form a p–n heterojunction, which is considered to increase the photocatalytic efficiency [6,11]. This heterojunction offers several advantages that can be attributed to CuO.

1. CuO provides a favorable bandgap energy range of 1.2–2.2 eV, which expands light absorption to the visible spectrum [5,7].
2. Coupling TiO_2_ with a lower-bandgap semiconductor can prolong its electron–hole separation lifetime by suppressing electron–hole recombination [5,6]. This prolonged separation facilitates the generation of hydroxyl radicals and is therefore critical to photodegradation.
3. The addition of H_2_O_2_, widely known as the Fenton process in AOPs, increases the production of hydroxyl radicals. Cu-based materials possess Fenton-like characteristics and can be used to degrade organic pollutants effectively in wastewater treatment [12].

The fabrication of CuO-loaded TiO_2_ nanomaterials has been investigated for applications such as hydrogen production [2,7,13], gas sensing [14], and pollutant degradation via photocatalysis [6,15,16]. However, conventional fabrication methods involve liquid-phase synthesis techniques, such as sol–gel processes [5,17] and hydrothermal methods [18] to obtain TiO_2_, or the direct use of commercially available TiO_2_ powder [8,19], which is subsequently loaded with CuO. However, liquid-phase synthesis involves several steps, potentially generates liquid waste, and is generally limited to small-scale production under laboratory conditions. 

These limitations in nanomaterial production can be addressed through gas-phase preparation. In our previous study [20], TiO_2_ nanoparticles were prepared in the form of thin films via plasma-enhanced chemical vapor deposition (PECVD). This process enables the creation of porous and well-dispersed nanoparticulate thin films, resulting in a larger active surface area, which enhances mass transfer in the nanomaterial and promotes the adsorption of organic pollutants. Moreover, for photocatalytic applications, immobilized nanoparticles or thin films are more advantageous than powdered nanomaterials because they eliminate the additional steps in catalyst recovery and facilitate reuse. In addition, by including an evaporation–condensation physical vapor deposition (PVD) system, CuO can be loaded onto TiO_2_ nanoparticles in a simple, one-step process.

As mentioned previously, the introduction of H_2_O_2_ has a positive effect on photocatalytic degradation by enhancing the photocatalytic activity. H_2_O_2_ serves as an electron scavenger, exhibiting high efficiency in generating hydroxyl radicals [21,22] and extending the light absorption range of TiO_2_ to include visible light [23]. In addition to studying the photocatalyst and its properties, other factors must be considered within the photocatalytic degradation system, including pH, which can markedly influence the efficiency and effectiveness of degradation [22,24].

This study was focused on the characteristics and performance of TiO_2_ and TiO_2_–CuO nanoparticulate thin films prepared via a one-step gas-phase process in a PECVD–PVD system, which has not been extensively reported. The performance of the fabricated films was determined by the photodegradation of a model dye pollutant rhodamine 6G (R6G) under UV and visible light irradiation. Moreover, the improvement in the photodegradation system with the addition of H_2_O_2_ and the effect of the pH on photodegradation were investigated to understand the optimized photodegradation conditions more fully.

## 2. Materials and Methods

### 2.1. Preparation of Nanoparticulate Thin Films

**Figure 1:**
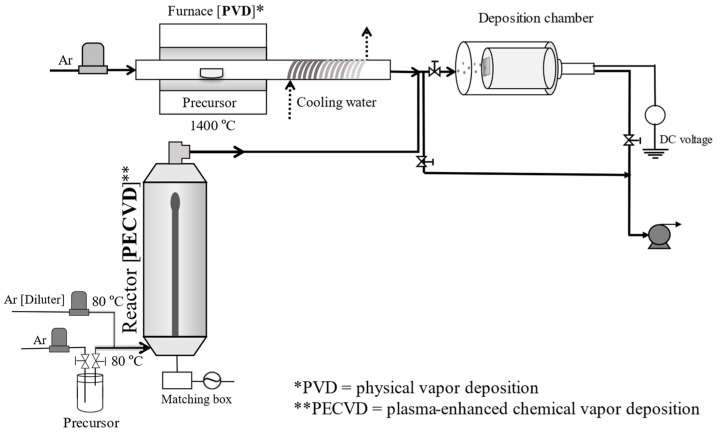
Schematic of the experimental setup.

The system employed for the formation of TiO_2_ and TiO_2_–Cu*_x_*O nanoparticulate thin films is shown in Figure 1. PECVD was conducted based on the method described in our previous papers [20,25]. Initially, the precursor titanium tetraisopropoxide (TTIP; Kanto Chemical Co., Inc., Tokyo, Japan) was fed into the plasma chamber with a radio frequency power supply (AX-1000IIP; Adtec Plasma Technology Co., Ltd., Fukuyama, Japan) at 13.56 MHz and 100 W. Ar was used as the carrier gas for the system, with a bubbling flow rate of 40 sccm and diluter flowrates of 350 and 100 sccm when the PVD system was in operation and idle, respectively. The TTIP molecules were bombarded by plasma to form TiO_2_ nanoparticles. Cu*_x_*O nanoparticles were formed in the PVD system by the evaporation of Cu chips (99.9% Cu, Nacalai Tesque, Inc., Kyoto, Japan) placed on the center of a tube inside a furnace (FT-01VAC-1630, Full-Tech Co., Ltd., Osaka, Japan) at 1400 °C. The vaporized Cu was transported by Ar at a flow rate of 900 sccm, and then rapidly cooled using a cooling water system that was coiled to the outer surface of the tube. For pristine samples of either TiO_2_ or Cu*_x_*O, the PECVD and PVD systems were operated separately. TiO_2_–Cu*_x_*O deposition was conducted in a one-step process; both the PECVD and PVD systems were operated simultaneously, and both streams of nanoparticles were deposited onto a Si substrate (1 × 1 cm^2^) at an applied voltage of +500 V. After deposition, the film was annealed in air at 500 °C for 3 h at a heating rate of 5 °C min^−1^ to oxidize Cu*_x_*O and crystallize the TiO_2_ nanoparticles completely. The weight of the fabricated films was maintained at approximately 0.08 mg.

### 2.2. Characterization of Nanoparticulate Thin Films

TiO_2_, Cu*_x_*O, and TiO_2_–Cu*_x_*O samples were characterized under different final conditions. The pristine TiO_2_ and TiO_2_–Cu*_x_*O samples were annealed at 500 °C, and the pristine Cu*_x_*O samples were as deposited. Scanning electron microscopy (SEM; S-5200, Hitachi High Technologies, Tokyo, Japan) was performed to examine the surface morphology of the nanoparticulate thin films. Transmission electron microscopy with energy dispersive X-ray spectroscopy (TEM-EDS; JEM-2010, JEOL, Tokyo, Japan) was conducted to observe the nanoparticle’s morphology and the elements within the film.

The crystallinity was confirmed using X-ray diffraction (XRD; MiniFlex 600, Rigaku, Tokyo, Japan) over the 2θ range of 20–60°, with Cu Kα (λ = 0.154 nm) radiation, an accelerating voltage of 40 kV, and a current of 15 mA. The chemical states were determined using X-ray photoelectron spectroscopy (XPS; ESCA-3400, Shimadzu Corp., Kyoto, Japan). Additionally, the reflectance was measured by UV–vis diffuse reflectance spectroscopy (UV-Vis DRS, V-650, Jasco, Tokyo, Japan). Zeta potential measurements (ZEN3690, Malvern Instruments Ltd., Malvern, UK) were also conducted.

### 2.3. Photocatalytic Tests

To assess the effectiveness of both pristine TiO_2_ and TiO_2_–Cu*_x_*O nanoparticulate thin films, R6G (Nacalai Tesque, Inc., Kyoto, Japan) was employed as a model pollutant. The deposited photocatalyst was submerged in a cuvette containing 3 mL of R6G aqueous solution at a concentration of 5 ppm. Additionally, to determine the effect of light, the experiment was conducted under UV irradiation (λ = 365 nm; AS ONE) and visible light irradiation (100 mW cm^−2^, λ > 385 nm; HAL-C100, Asahi Spectra, Tokyo, Japan). Furthermore, for several samples, a single drop (~0.05 mL) of diluted H_2_O_2_ (Kanto Chemical Co., Inc., Tokyo, Japan) was added to the 3 mL R6G aqueous solution; consequently, the final H_2_O_2_ concentration was 22.3 mM. The photocatalytic activity was also tested under acidic (pH 3) and basic conditions (pH 13) with the addition of HNO_3_ (Nacalai Tesque, Inc., Kyoto, Japan) and NaOH (Fujifilm Wako Pure Chemical Corporation, Tokyo, Japan), respectively. The photocatalytic performance of the fabricated film under UV irradiation was compared with that of commercially available TiO_2_ (P25, Sigma–Aldrich, Burlington, MA, USA) nanoparticles immobilized using the doctor-blade method. The fabricated film and immobilized P25 weighed 0.08 mg each, with an area of 1 × 1 cm^2^, which was the size of the Si substrate.

For the photocatalytic test, the samples were initially placed in the dark for 30 min at a controlled temperature of 25 °C. Subsequently, the dye photodegradation was monitored in 30 min intervals for 3 h. To assess the degree of dye degradation, changes in the absorbance of R6G at 530 nm were measured using the UV–vis spectrophotometer. According to the Beer–Lambert law, the change in concentration was proportional to the change in absorbance at 530 nm. The efficiency of dye degradation is subsequently represented by the normalized concentration (C*_t_*/C_0_), which is the ratio of the dye concentration at time *t* to the initial concentration.

## 3. Results and Discussion

### 3.1. Characteristics

**Figure 2:**
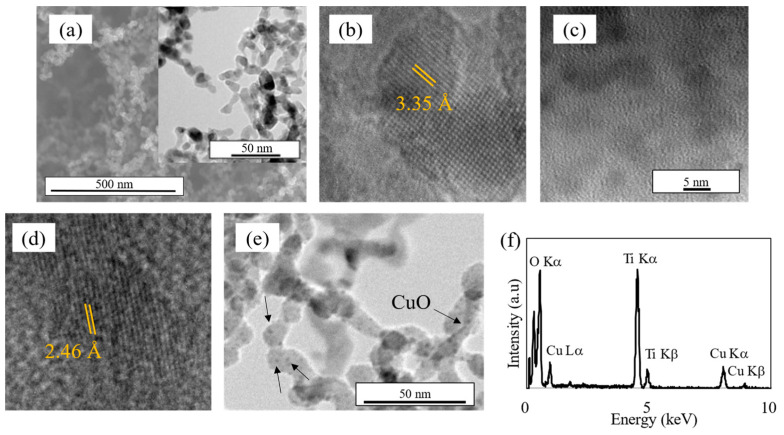
Nanoparticle morphology. (**a**) SEM images of TiO_2_ (inset: TEM). HR-TEM images of (**b**) TiO_2_ and (**c**,**d**) Cu*_x_*O. (**e**) TEM image and (**f**) EDS spectrum of TiO_2_-CuO.

The SEM image of pristine TiO_2_ in Figure 2a reveals the surface morphology of the annealed thin film, consisting of nanoparticles. The observed structure is consistent with the findings of our previous study [20], indicating that a highly porous film was formed with minimal agglomeration. This porous, low-agglomeration film is significant for photocatalytic applications, owing to its increased surface-to-volume ratio, which can enhance the effectiveness of photodegradation, with a higher active surface area for dye adsorption on the nanoparticle surface.

Further insight into the morphology was obtained through TEM analysis (Figure 2a–e). The pristine TiO_2_ and Cu*_x_*O nanoparticles were observed to be spherical, with sizes of 7–15 and 8–14 nm, respectively. Based on the TEM results for pristine TiO_2_ (Figure 2a,b) and TiO_2_–Cu*_x_*O (Figure 2e), the introduction of Cu*_x_*O did not appear to alter the morphology of the TiO_2_ nanoparticles. Careful observation of the TiO_2_–Cu*_x_*O samples (Figure 2e) revealed that Cu*_x_*O nanoparticles were deposited on the TiO_2_ nanoparticle surface, confirming the existence of Cu*_x_*O in the film. The EDS analysis results further confirmed the presence of Cu*_x_*O (Figure 2d). The EDS spectrum depicted peaks corresponding to Cu La, Cu Ka, Cu Kb, Ti Ka, Ti Kb, and O Ka, confirming the existence of Cu*_x_*O and TiO_2_. Therefore, TiO_2_–Cu*_x_*O nanoparticulate thin films were successfully fabricated via the one-step gas-phase deposition.

**Figure 3:**
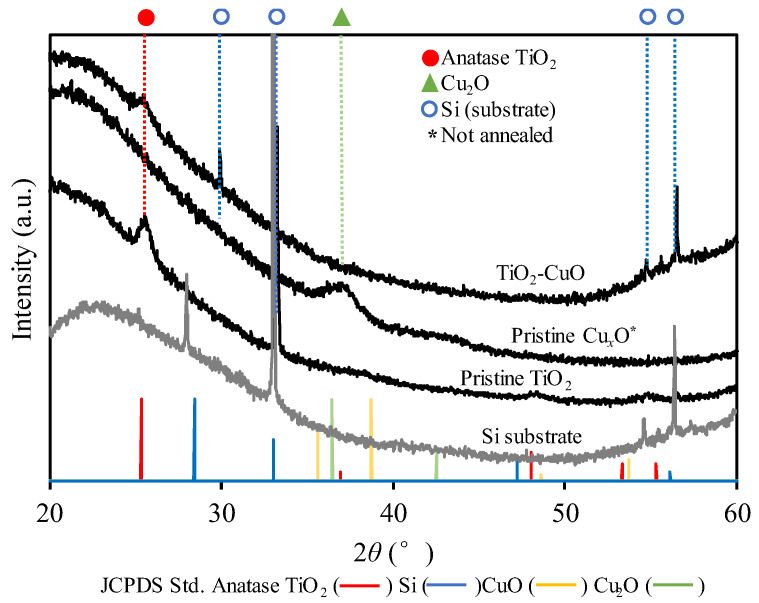
XRD patterns indicating the crystallinity of the fabricated nanoparticulate thin films.

Crystallinity is a pivotal characteristic that influences photocatalytic activity. Based on the high-resolution TEM findings in Figure 2b,d, the crystallization of the pristine TiO_2_ post-annealing and the as-deposited pristine Cu*_x_*O can be confirmed by the measured interplanar spacings of 3.35 and 2.46 Å, respectively. In addition, the crystallization can be confirmed by the XRD analysis results. For TiO_2_, the photocatalytic activity of the anatase crystalline phase was evidently higher than that of the rutile phase. The XRD pattern confirmed the crystalline nature of TiO_2_ nanoparticles after annealing; the peak of anatase TiO_2_ was observed at approximately 2θ = 25° (JCPDS no. 21-1272) (Figure 3). For pristine Cu*_x_*O nanoparticles, the visible peak corresponded to Cu_2_O at 2θ = 37.16° (JCPDS no. 5-0667), confirming the crystalline state of the nanoparticles upon deposition onto the substrate even before annealing. However, the peaks from the TiO_2_–Cu*_x_*O nanocomposite sample represented only anatase TiO_2_, and no visible peaks corresponding to Cu_2_O or CuO were observed. Furthermore, no shift was observed in the peaks upon the addition of Cu*_x_*O to TiO_2_. The absence of Cu*_x_*O peaks could be attributed to the relatively low concentration and dispersion of Cu*_x_*O loaded [13]. The crystallite size of the nanoparticles could be estimated from Scherrer’s equation *D* = *kλ*/(*B*cos*θ*), where *k*, *λ*, *B*, and *θ* denote the constant value (*k* = 0.89), wavelength of the X-ray source (*λ* = 0.154 nm), full-width at half-maximum (FWHM) of the XRD peak, and peak angle, respectively [26]. The calculation yielded crystallite sizes of 13.07 nm for pristine TiO_2_ and 14.99 nm for TiO_2_-CuO, which are reasonable compared to the sizes obtained from direct measurement of the nanoparticles from the TEM images.

**Figure 4:**
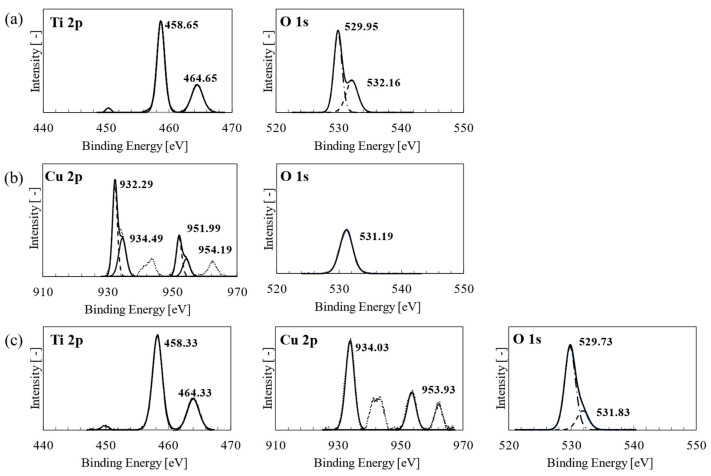
XPS spectra of (**a**) pristine TiO_2_, (**b**) pristine Cu_x_O, and (**c**) TiO_2_–Cu*_x_*O nanoparticulate thin films. The solid and dotted lines represent the XPS and deconvoluted spectra, respectively.

The chemical composition of the nanoparticulate thin film was determined using XPS to confirm the presence of Cu*_x_*O and the effect of Cu*_x_*O on the chemical state of the TiO_2_ nanoparticles. In particular, the Ti 2p, O 1s, and Cu 2p core level spectra were used to determine the chemical composition and valence state on the surface of the film (Figure 4a–c). In the Ti 2p profile for pristine TiO_2_ (Figure 4a), the binding energies (BE) for the Ti 2p_3/2_ and Ti 2p_1/2_ peaks were 458.65 and 464.65 eV, respectively, corresponding to Ti^4+^ of TiO_2_ [27]. Similarly, in the Ti 2p profile for TiO_2_–Cu*_x_*O, BEs of 458.33 and 464.33 eV were observed for Ti 2p_3/2_ and Ti 2p_1/2_, respectively. The O 1s spectrum revealed multiple peaks in the pristine TiO_2_ film. BEs of 529.96 and 532.16 eV were detected for lattice O (O_L_) and hydroxyl O, respectively. These corresponded to O binding with titania (Ti^4+^) and the surface hydroxyl group (-OH), respectively [27]. Pristine Cu*_x_*O exhibited a higher BE (531.19 eV) for O_L_ in the O 1s spectrum, which was attributed to the variation in Cu or Ti bonding to O. In TiO_2_–Cu*_x_*O, the BEs for O 1s were 529.73 and 531.83 eV, indicating a slight shift compared with that observed for pristine TiO_2_, attributed to the interaction between Cu ions and TiO_2_ [28]. For as-deposited pristine Cu*_x_*O, Cu 2p_3/2_ and Cu 2p_1/2_ peaks were detected at 932.29 and 951.99 eV, respectively, corresponding to Cu^+^ (Figure 4b). In addition, the BEs of 934.49 and 954.19 eV were associated with peaks corresponding to Cu^2+^. For the annealed TiO_2_–Cu*_x_*O film, the presented peaks were only ascribed to Cu^2+^ at Cu 2p_3/2_ and Cu 2p_1/2_, with BEs of 934.03 and 953.93 eV, respectively (Figure 4c). The satellite peaks of approximately 940 and 960 eV in both Cu 2p results (Figure 4b,c) corresponded to the existence of Cu^2+^. These results indicate that Cu was completely oxidized after annealing in air; this nanocomposite is subsequently referred to as TiO_2_–CuO.

**Figure 5:**
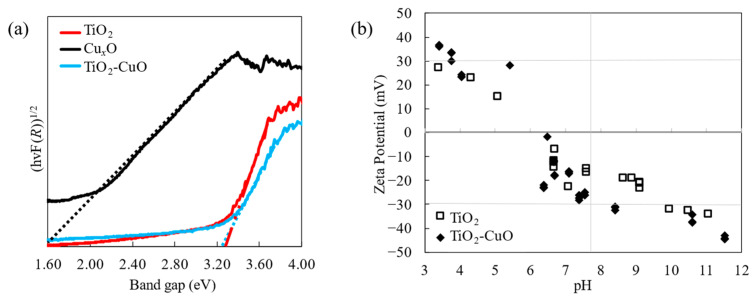
(**a**) Plot of (*hν*F(*R*))^1/2^ versus *hν* for the pristine TiO_2_, pristine Cu_x_O, and TiO_2_–CuO nanoparticulate thin films. (**b**) Zeta potentials of the pristine TiO_2_ and TiO_2_–CuO nanoparticles as a function of pH.

Figure 5a illustrates the bandgap energy of the film based on reflectance data obtained from UV–vis DRS measurements and the Kubelka–Munk function, which can be expressed as F(*R*) = (1 − *R*)^2^/2*R*, where *R* is the reflectance [3,29]. The band gap was estimated from the intercept of the (*hν*F(*R*))^1/2^ versus *hν* plot, where *h* denotes Planck’s constant (1240 eV) and *ν* denotes the frequency (eV). The bandgap energies of the pristine Cu*_x_*O, pristine TiO_2_, and TiO_2_-CuO nanoparticulate thin films were 1.6, 3.26, and 3.25 eV, respectively. Notably, the bandgap energy of TiO_2_–CuO was lower than that of pristine TiO_2_. However, only a slight decrease in the bandgap was observed because of the small amount of CuO added to the film. Nevertheless, the lower bandgap energy of TiO_2_ with added CuO was possible because of the charge transfer of the p–n heterojunction between TiO_2_ and CuO, as reported previously [30]. This result is consistent with the XPS results in Figure 4, where the decrease in the BE with the addition of CuO to TiO_2_ indicates a decrease in the bandgap energy.

Figure 5b shows a graph of the zeta potentials. The graph indicates that the TiO_2_ and TiO_2_–CuO samples had positively charged surfaces under acidic conditions and negatively charged surfaces under basic conditions. Zero zeta potential was observed at pH 6.5 for pristine TiO_2_, and similarly for TiO_2_–CuO, implying that the surface charge did not substantially change after CuO was added to TiO_2_. A negatively charged surface is advantageous for interactions with cationic dyes such as R6G. This charge configuration enhances the affinity of the nanoparticle surface of the film to the dye, which can increase the photocatalytic degradation, as reported previously [17,24].

### 3.2. Photocatalytic Degradation of R6G

#### 3.2.1. UV Irradiation

**Figure 6:**
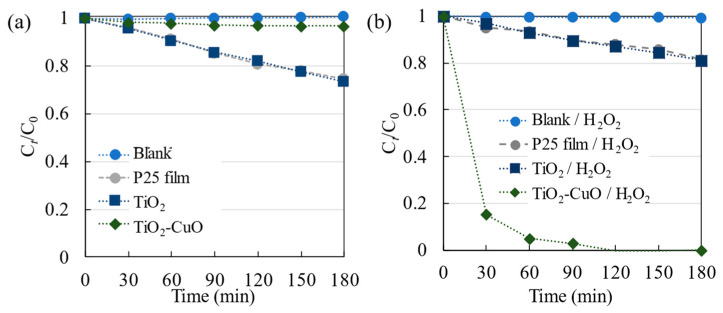
Photolysis and photocatalytic degradation of R6G on (**a**) pristine TiO_2_, TiO_2_–CuO, and P25 films; and (**b**) the corresponding samples with added H_2_O_2_ under UV irradiation.

The degradation of R6G through photocatalysis under UV irradiation was assessed by monitoring the absorbance of the dye using a UV–vis spectrophotometer. The normalized dye concentration was plotted as a function of time (Figure 6). The efficiency of direct photolysis (depicted as a blank) or dye degradation in the absence of a catalyst was limited, as C*_t_*/C_0_ was less than 0.99 within 180 min of irradiation. The addition of a TiO_2_ nanoparticulate film to the photodegradation system promoted the photodegradation of the dye rather than photolysis, resulting in C*_t_*/C_0_ = 0.73 after 180 min of irradiation. However, the photocatalytic activity decreased when the TiO_2_–CuO nanoparticulate thin film was used (C*_t_*/C_0_ = 0.96). CuO was introduced into the system as an electron acceptor to decrease the electron–hole recombination in TiO_2_; the results indicate that the presence of CuO potentially amplified charge recombination, effectively transforming CuO into a center for electron–hole recombination.

To enhance the photodegradation of the dye further, H_2_O_2_ (~0.05 mL; 22.3 mM) was introduced into the photodegradation system as an electron acceptor. The addition of H_2_O_2_ did not substantially increase the photolysis (Blank/H_2_O_2_) or photocatalytic (TiO_2_/H_2_O_2_) degradation of R6G, with C*_t_*/C_0_ values of 0.99 and 0.81, respectively (Figure 6b). However, the performance of TiO_2_ differed under visible light irradiation, as is elucidated in Section 3.2.2.

**Figure 7:**
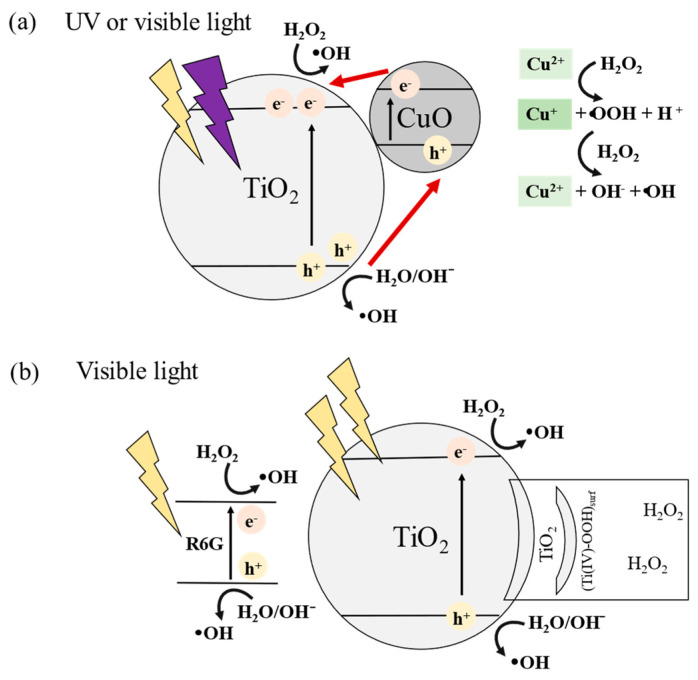
Photodegradation mechanism of (**a**) TiO_2_–CuO under UV and visible light irradiation and (**b**) pristine TiO_2_ under visible light irradiation.

Compared to the other films, photocatalytic degradation by the TiO_2_–CuO nanoparticulate thin film was considerably increased with the addition of H_2_O_2_, as R6G was completely degraded at 180 min. The possible degradation mechanism can be explained using Figure 7a for TiO_2_-CuO/H_2_O_2_. Following previous reports [12], a p-n heterojunction (type II) was formed by introducing CuO into TiO_2_, and light irradiation was employed to generate electron–hole pairs. The electrons excited from the valence band of CuO were transferred to the conduction band of TiO_2_. A Fenton-like reaction of Cu^2+^ (green blocks) and photo-generated electrons (pink circles) with H_2_O_2_ can effectively increase the production of hydroxyl radicals (⋅OH), which can improve the photocatalytic activity [12]. In addition, the generated holes will react with adsorbed water (H_2_O) or surface hydroxyl (OH^−^), forming ⋅OH [1,16]. A comparison of the results in Figure 6a,b indicates a substantial improvement in the photocatalytic activity when H_2_O_2_ and CuO were added.

To assess the effectiveness of the fabricated TiO_2_ nanoparticulate thin film, its photocatalytic activity was compared with that of the P25 film. The R6G photodegradation rates of the fabricated TiO_2_ nanoparticulate thin film and P25 film were similar, regardless of the presence of H_2_O_2_. This consistency highlights the effectiveness of the photocatalytic activity of the film produced in this study.

#### 3.2.2. Visible Light Irradiation

**Figure 8:**
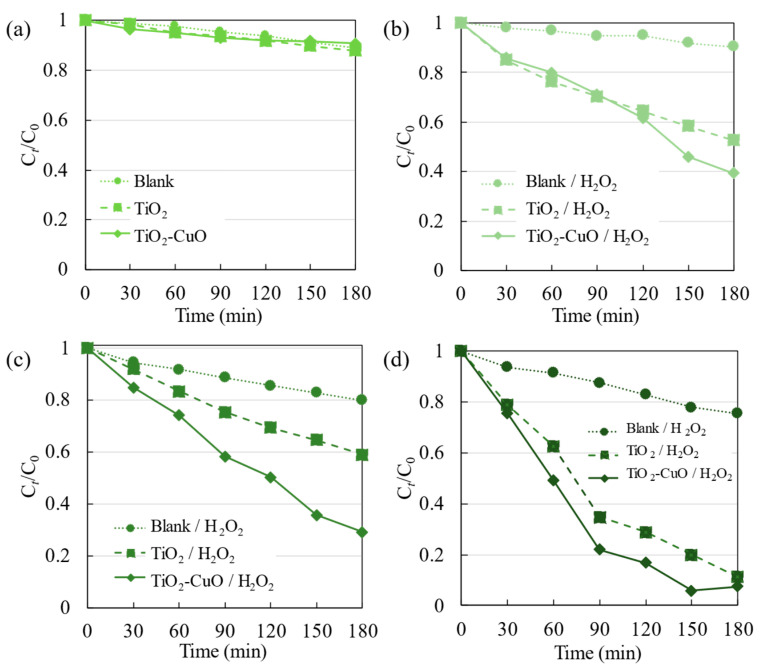
Photolysis and photocatalytic degradation of R6G on pristine TiO_2_, TiO_2_–CuO, and samples with added H_2_O_2_ under visible light irradiation (λ > 385 nm) at pH values of (**a**,**b**) 7, (**c**) 3, and (**d**) 13.

In photocatalytic applications, TiO_2_ generally exhibits limited activity under UV irradiation. Therefore, a photocatalytic activity test was performed in the visible light region to evaluate the produced film and its degradation conditions. Considering the varying pH levels of wastewater effluents, the pH of the photodegradation system was adjusted to values of 3 or 13. The results are shown in Figure 8.

When H_2_O_2_ was not added, pristine TiO_2_ and TiO_2_–CuO demonstrated low activity for the photolysis and photocatalytic degradation of R6G, with the same value of C*_t_*/C_0_ (0.90) within 180 min of irradiation (Figure 8a). The addition of the photocatalyst did not improve photodegradation under visible light irradiation. Moreover, compared with the R6G photolytic activity observed under UV irradiation, R6G can possibly undergo self-degradation when irradiated with visible light.

Figure 8b presents the photodegradation with the addition of H_2_O_2_. The photolysis of R6G only increased slightly with the addition of H_2_O_2_. Moreover, the photocatalytic degradation of R6G by pristine TiO_2_ and TiO_2_–CuO film samples resulted in C*_t_*/C_0_ values of approximately 0.52 and 0.39, respectively. Photodegradation by TiO_2_/H_2_O_2_ was considerably more effective under visible light than under UV irradiation. Figure 7b illustrates the condition of the TiO_2_ surface with added H_2_O_2_ under visible light irradiation. The addition of H_2_O_2_ altered the TiO_2_ surface, enabling visible light absorption owing to the newly formed surface complexes of TiO_2_ [23]. Furthermore, as depicted in Figure 7b, the photosensitization of R6G under visible light irradiation in the presence of a semiconductor was able to increase the formation of electrons and holes [31], thereby promoting photodegradation. The higher activity of TiO_2_–CuO/H_2_O_2_, as was also observed under UV irradiation, can be attributed to the Fenton-like reaction resulting from the involvement of H_2_O_2_ in the photocatalytic degradation system. Therefore, the addition of H_2_O_2_ to the TiO_2_–CuO system was considerably more effective for the photodegradation of R6G (Figure 7a).

**Figure 9:**
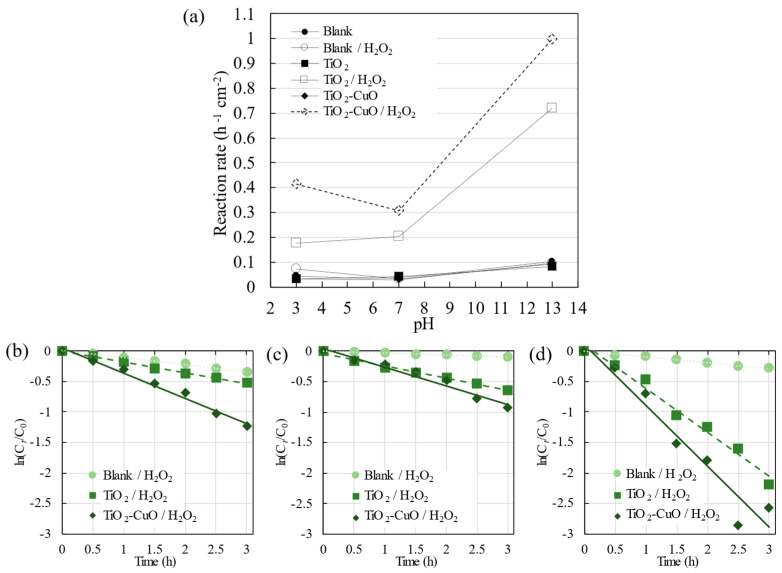
(**a**) Pseudo-first-order rate constant for R6G photodegradation under visible light irradiation, calculated from the slope of ln(C*_t_*/C_0_) as a function of time; representative curves of ln(C*_t_*/C_0_) versus time for samples at pH values of (**b**) 3, (**c**) 7, and (**d**) 13.

The R6G dye had a pH of 7. To investigate the impact of different pH conditions, acidic (pH 3) and basic (pH 13) conditions were attained by adding HNO_3_ and NaOH, respectively, to R6G. Comparing the photolysis results depicted in Figure 8b–d (Blank/H_2_O_2_) reveals that the activity increased in the order pH 3 > 13 > 7. At a lower pH, where HNO_3_ was added to the photodegradation system, the photolysis of R6G increased. According to a previous report [32], the increased activity under either acidic or basic conditions is governed by different mechanisms, and degradation is enhanced under both conditions. Nevertheless, when the photocatalyst was not employed, photodegradation by H_2_O_2_ under varying pH conditions remained relatively insignificant, in contrast to the significant degradation observed upon the addition of the photocatalyst (Figure 9). This characteristic demonstrates the relevance of dye degradation via the photocatalytic mechanism.

The reaction rate constant (Figure 9a) was calculated using the slope of ln(C*_t_*/C_0_) = *kt*, where C_0_, C*_t_*, *t*, and *k* denote the initial concentration, concentration at a given time, time (h), and reaction rate constant (h^−1^ cm^−2^), respectively [25]. Furthermore, for the calculation of *k*, the relationship between ln(C*_t_*/C_0_) and *t* is depicted in Figure 9b–d, with the photocatalytic activity within 180 min (3 h) of light irradiation. These results indicate that the degradation of R6G was notably enhanced through the use of the photocatalyst and the addition of H_2_O_2_. TiO_2_–CuO/H_2_O_2_ demonstrated the highest degradation of R6G under visible light irradiation across different pH levels, with the highest value at pH 13 and a reaction rate constant of 0.99 h^−1^ cm^−2^. The photodegradation of R6G by pristine TiO_2_/H_2_O_2_ and TiO_2_–CuO/H_2_O_2_ films was more effective than the photolysis of R6G by H_2_O_2_ (Blank/H_2_O_2_) under visible light irradiation. The photocatalytic activity of TiO_2_/H_2_O_2_ followed the order of pH 13 > 7 > 3, which is consistent with the zeta potential results. Under basic conditions, the fabricated film possessed a negatively charged surface, thereby exhibiting a higher potential for adsorbing R6G than under acidic conditions, where the surface was positively charged. These interactions enhanced the efficiency of photodegradation. This finding indicates that photodegradation strongly depends on the surface conditions of the photocatalyst.

TiO_2_–CuO/H_2_O_2_ exhibited higher photocatalytic activity across all pH values compared to both photolysis with the addition of H_2_O_2_ and the photocatalytic activity of TiO_2_/H_2_O_2_; this improvement can be attributed to the Fenton-like reaction occurring with the addition of CuO. Further observation revealed that the photocatalytic activity followed the order of pH 13 > 3 > 7. Notably, the photodegradation was substantially enhanced at pH 13 when TiO_2_–CuO was used instead of pristine TiO_2_. The increased activity of TiO_2_–CuO at pH 13 compared to the other pH values can be attributed to the adsorption of R6G on TiO_2_. Furthermore, the increase in activity at pH 3 can be attributed to the TiO_2_–CuO interaction and the Fenton-like reaction of CuO and H_2_O_2_. Additionally, the presence of H^+^ ions at pH 3 increased the production of hydroxyl radicals, which degrade R6G.

## 4. Conclusions

In this study, a one-step gas-phase approach using PECVD and PVD was successfully employed to fabricate porous and low-agglomeration pristine TiO_2_ and TiO_2_–CuO nanoparticulate thin films. The resulting film comprised small, spherical TiO_2_ nanoparticles, with CuO observed on the surface of TiO_2_ upon loading. In addition, annealing in air resulted in the crystallization of the TiO_2_ nanoparticles and complete oxidization of CuO. Under UV irradiation, the photolysis and photodegradation of R6G by pristine TiO_2_ resulted in degradation values (C*_t_*/C_0_) of 0.99 and 0.81, respectively. The addition of H_2_O_2_ to the TiO_2_–CuO film was considerably more effective, as R6G was completely degraded. Under visible light irradiation, the addition of H_2_O_2_ was effective for the photolysis of R6G and its photocatalytic degradation by TiO_2_ and TiO_2_–CuO, with degradation values of 0.90, 0.52, and 0.39, respectively. Furthermore, the influence of the pH on the photocatalytic activity was evident; the highest activity was exhibited by TiO_2_–CuO/H_2_O_2_ at pH 13, with a reaction rate constant of 0.99 h^−1^ cm^−2^ under visible light irradiation. The enhanced performance of TiO_2_–CuO/H_2_O_2_ is promising for wastewater treatment and purification applications.

The present study focused on the one-step gas phase fabrication of TiO_2_ and TiO_2_–CuO nanoparticulate thin films and their application to the photodegradation of R6G under UV and visible light irradiation. However, further studies are necessary to comprehensively evaluate the active species involved and the reaction pathways in addition to changes in the overall organic content during photocatalysis. These research areas provide opportunities to optimize the experimental conditions and expand the application scope.

## Data Availability

Data are contained within the article.
